# Improving Equity in Urban Immunization in Low- and Middle-Income Countries: A Qualitative Document Review

**DOI:** 10.3390/vaccines11071200

**Published:** 2023-07-04

**Authors:** Rachel Victoria Belt, Shakil Abdullah, Sandra Mounier-Jack, Samir V. Sodha, Niklas Danielson, Ibrahim Dadari, Folake Olayinka, Arindam Ray, Tim Crocker-Buque

**Affiliations:** 1 Nuffield Department of Women’s and Reproductive Health, University of Oxford, 75 George Street, Oxford OX1 2RL, UK; 2 Department of Anthropology, University of Connecticut, Storrs, CT 06269, USA; 3 Department of Global Health Development, London School of Hygiene and Tropical Medicine, London WC1E 7HT, UK; 4 Department of Immunization Vaccines and Biologicals, WHO Headquarters, Avenue Appia 20, 1202 Geneva, Switzerland; 5 Coverage & Equity Unit, Immunization Section, PG-Health, UNICEF Headquarters, 3 UN Plaza, New York, NY 10017, USA; 6 Public Health Institute, STAR Fellow Department, 901 D St, SW, Suite 1040, Washington, DC 20024, USA; 7 Bill & Melinda Gates Foundation, New Delhi 110075, India

**Keywords:** vaccines, immunization, urban health, equity, health systems, health planning, routine immunization, zero-dose, urban immunization

## Abstract

Introduction: As the world continues to urbanize, particularly in low- and middle-income countries, understanding the barriers and effective interventions to improve urban immunization equity is critical to achieving both Immunization Agenda 2030 targets and the Sustainable Development Goals. Approximately 25 million children missed one or more doses of the diphtheria, tetanus and pertussis (DTP3) vaccine in 2021 and it is estimated that close to 30% of the world’s children missing the first dose of DTP, known as zero-dose, live in urban and peri-urban settings. Methods: The aim of this research is to improve understanding of urban immunization equity through a qualitative review of mixed method studies, urban immunization strategies and funding proposals across more than 70 urban areas developed between 2016 and 2020, supported by Gavi, the Vaccine Alliance. These research studies and strategies created a body of evidence regarding the barriers to vaccination in urban settings and potential interventions relevant to low- and middle-income countries (LMICs) with a focus on the vaccination of urban poor, populations of concern and residents of informal settlements. Through the document review we identified common challenges to achieving equitable coverage in urban areas and mapped proposed interventions. Results: We identified 70 documents as part of the review and categorized results across (1) social determinants of health, (2) immunization service-delivery barriers and (3) quality of services. Barriers and solutions identified in the documents were categorized in these thematic areas, drawing information from results in more than 21 countries. Conclusion: Populations of concern such as migrants, refugees, residents of informal settlements and the urban poor face barriers to accessing care which include poor availability and quality of service. Example solutions proposed to these challenges include tailored delivery strategies, improved use of digital data collection and child-friendly services. More research is required on the efficacy of the proposed interventions identified and on gender-specific dynamics in urban poor areas affecting equitable immunization coverage.

## 1. Introduction

As of 2019, an estimated 55% of the world’s population lives in urban areas and this percentage is expected to increase to 68% by 2050 [1]. Approximately 90% of the projected increase will occur in Asia and Africa [1]. It is also expected that this trend, especially in LMICs, will be linked to population growth in informal settlements defined by the UN Habitat as meeting four out of five household shelter deprivations: lack of access to improved water, sanitation, sufficient living area, durability of housing structures and insecurity of tenure [2]. Globally, vaccination coverage rates appear higher in urban than in rural areas—the so-called urban advantage; however, this trend is reversing as countries become more urbanized. Large numbers of zero-dose and under-immunized children reside in urban areas, an estimated 30% of zero-dose children, globally [3]. Additional information is required on the barriers for urban informal settlements, particularly for populations of concern and to identify solutions which can improve urban immunization equity. This review draws on a variety of country-specific sources to analyze the latest research and program designs in the urban context.

Gavi, the Vaccine Alliance is a global financing entity for vaccination with a strategic priority on improving the equity of vaccination to reach zero-dose children. The Alliance includes the World Health Organization (WHO), UNICEF, the World Bank, the Bill and Melinda Gates Foundation (BMGF), the US Centers for Disease Control (CDC), implementing country governments and civil societies [4]. To inform Gavi financing investments and country-specific research priorities, Gavi supports governments and technical partners in developing urban immunization research studies to identify challenges to urban immunization equity. Often drawing on the same urban immunization studies, governments developed city-specific strategies and proposals for Gavi funding investments which included proposed solutions and program designs for urban informal settlements and populations of concern. The research and strategy documents are the focus of this documentary review, which aims to understand the barriers to vaccination in the urban context and potential interventions. The study objectives were to (1) distill common findings and lessons learned from the urban diagnostics and costed strategies developed between 2016 and 2020 for urban immunization programming and (2) identify research questions for further evaluation of urban immunization approaches and priority areas for implementation research. The research questions are available in Table 1 and span socio-economic determinants of health, service delivery, and demand generation. This research aims to contribute to the World Health Organization’s “Global Research Priorities for Urban Health”, including the study of underrepresented groups [5].

The research plan and coding framework were developed in consultation with the global Urban Immunization (UI) working group. The UI working group was created by key immunization partners, including UNICEF and WHO, to create awareness about inequities in urban areas and support development of strategies to meet specific immunization needs of marginalized communities.

## 2. Methods

We used a qualitative content-analysis methodology to analyze results across country urban immunization diagnostic studies, presentations, strategic documents and proposals. Diagnostic studies were mixed methods, including qualitative and quantitative data on urban immunization. Strategy documents were developed by technical partners and Ministries of Health to plan and cost interventions to address identified barriers to immunization coverage. The investment proposals were funding applications for Gavi support from Ministries of Health. Proposals which included urban immunization were eligible in the review as they presented data on urban equity and interventions designed to improve urban immunization. In this research, we followed a three-step content analysis process proposed by Elo and Kyngas (2008): 1. Preparation, 2. Organizing, and 3. Reporting [6].

In the preparation phase, we sourced and identified the prospective documents for analysis. To source documents, a request for urban diagnostics, strategies, and proposals was shared with all country focal points at Gavi, the Vaccine Alliance in 2019 and early 2020. All shared documents were organized into a digital Sharepoint library inclusive of government, technical assistance, and internal proposals. All documents were reviewed against the criteria in Table 2 for inclusion in the analysis. Included country urban immunization documents are shared in the Supplementary Materials. We screened the documents for eligibility against the criteria. The selected documents were in three languages, English, French, and Portuguese. Due to the language skills of the researchers, the English and French documents were coded in their original language. Portuguese documents from Angola were translated through Google Translator, an online free translation service. Budget documents were deemed ineligible for this analysis as the budgets were not permitted for wide circulation. In the organizing phase, a coding framework was developed using the Urban Immunization Toolkit, a guidance for urban immunization planning, and refined in consultation with experts from the UI working group [7]. In this phase, we also prioritized the recurring themes and sub-themes from the preliminary reading of the documents with the support of the UI working group. A reviewer uploaded the documents to a Sharepoint library and used NVIVO software (Release 1.0) by QSR International, a computer software program, to organize and analyze qualitative data. The diagnostic research documents, country urban immunization strategies, and investment proposals were coded using both deductive and inductive approaches in line with the coding framework shared in the supplementary documents. The framework aligned with the key thematic areas (1) urban environment (socio-economic determinants of health), (2) service delivery and (3) demand interventions (quality of care), (4) gender barriers and (5) solutions. All documents, which were either in PDF or Word format, were coded similarly, regardless of type. In the reporting phase, the second reviewer analyzed the results of the coding in line with the identified themes and drafted the analysis which continued to follow the original thematic areas in the coding framework. The findings were presented to the UI working group for discussion, revision and validation.

## 3. Results

Among the 70 documents included in the analysis, there were 30 diagnostic studies, 1 study presentation, 16 strategies, 6 strategy presentations, 14 funding proposals, 2 application review documents, and a meeting report. Forty-six of the documents were dated 2018 and 2019, with 10 documents having an unknown date. The source of the data in the documents varied, with some studies engaging directly with marginalized communities through Focus Group Discussions (FGDs) and others drawing inferences from existing data sets. All documents focused on the urban context.

The studies and strategies across the urban immunization programs used both quantitative and qualitative methodologies to identify and measure immunization barriers. There are four data points or sources in the studies: (1) assessing health facilities and mapping (quantitative data), (2) caregiver exit interviews (qualitative data), (3) interviews with the frontline workers and caregivers (qualitative data including focus group discussions and key informant interviews), and (4) solutions and recommendations for improvement. Strategies detailed prioritized interventions for improving immunization equity in urban areas.

The urban immunization strategies and financing proposals included information on barriers to vaccination and proposed interventions. We identified and included reports and costed strategies across 20 Gavi-eligible countries to understand the specific challenges to equitable immunization coverage in selected cities. To analyze the findings, we used a thematic analysis derived from the urban immunization toolkit organized by (1) the social and economic determinants of health, (2) immunization service delivery, and (3) the quality of service.

### 3.1. Social and Economic Determinants of Health

#### 3.1.1. Identified Challenges Related to Social and Economic Determinants of Health

The diagnostic reports and country strategies presented evidence of inequalities in service delivery to populations of concern, such as migrants, refugees, internally displaced persons, and homeless and poor populations in urban contexts such as residents of informal settlements. Inequities included availability and proximity to service, discrimination, and security. Table 3 summarizes the populations of concern and their associated barriers from the review.

Populations of concern may have to travel far for services and may not be included in microplanning for outreaches. In a study in Somalia on three urban centers, IDP caregivers reported that they were not part of the immunization campaign, even though they expressed their desire to have access to immunization services [8]. Proximity of vaccination services to camps is a barrier to displaced communities. In Hargeisa, Somalia, IDP camps are far away from health facilities; two out of ten facilities had microplanning to reach out to IDP communities, whereas the remaining eight others had no such plan or strategy in place. Similarly, in Afghanistan, four of ten camps were mapped and included in microplanning activities, while others were located out of the city. Data showed that the three districts in Kabul with three IDP camps have no health facility [9].

Marginalized communities, such as refugees and populations with insecure housing face additional barriers to reaching services, including the lack of residency cards for publicly supported services and language barriers. Refugees were one group which experienced discrimination and inequality in accessing immunization services for marginalized populations in urban settings. In Uganda, refugees reported being skipped in the waiting line and charged additional fees. Security concerns also present a unique challenge for health workers and caregivers alike. In Cite-Soleil, Haiti, 18% cited fear of insecurity as a reason for incomplete vaccination, “zero dose cases in slums are mostly due to vaccinators’ reluctance to visit security risk areas” [10].

#### 3.1.2. Identified Solutions to Improve Barriers Related to Social and Economic Determinants of Health

Solutions aimed to tailor delivery and demand strategies to reach marginalized urban residents through community engagement and improved forecasting of vaccines to populations of concern. Recommendations included in country diagnostics and strategy reports are summarized in Table 4, including mobile and outreach strategies (market, street, places of worship), improved training of health workers on interaction and information sharing, and tracking of migrant families to avoid loss to follow-up.

### 3.2. Immunization Service Availability 

#### 3.2.1. Identified Challenges to Service Availability

Service-delivery barriers facing urban communities related to the provision, distribution, and quality of the major inputs of immunization services such as (1) health workers (vaccinators and community health workers), (2) immunization site location, accessibility, and hours (clinics, outreaches), (3) functional cold chain equipment, and (4) vaccine availability due to poor population data, last mile stock visibility and forecasting, and the incorrect application of open-vial policy. Moreover, there were significant governance issues between the government ministries, and private providers. A summary of common challenges across these areas is provided in Table 5.

Insufficient sites for immunization services may result in long travel times. In Kabul, 69% of the population takes over one hour to reach a health facility [9]. In Pakistan’s urban informal settlements, 28% of mothers surveyed cited that government health facilities were too far away [11]. In Uganda and Bangladesh, the proximity of services was high, although largely private [12]. Despite the effort to ensure quality of immunization services, there were gaps in services when compared to national immunization guideline requirements, especially in relation to cold chain and vaccine management, recording and reporting, injection safety, management of adverse events, and waste management.

Kenya, Ghana, Haiti, and Uganda’s urban assessments noted stock-outs as a barrier to vaccination [10,13,14]. In Nairobi and Kisumu, Kenya, 20% and 14% of facilities, respectively, reported having experienced a vaccine stock-out in the 3 months preceding the urban assessment [14]. Stock-outs also affected demand for immunization in urban areas. A civil society representative, interviewed in Ghana noted, “I have realized this [vaccine shortage] on two occasions and when this happens the mothers do not come again because they have a problem with transportation” [13].

Public service-delivery provision is not expanding at the rate of the rapid population growth in urban areas and results in many of the issues described above such as stock-outs and long wait times. The gap in public service delivery is often filled by private and faith-based facilities. This creates a challenge in coordinating, managing, and maintaining immunization service quality. In Nepal, the reasons for choosing the facility by clients included proximity of residence (70%) and perceived better-quality services (37.5%) [15]. In Kyrgyzstan, caregivers had mixed experiences concerning the different types of immunization providers. Some caregivers favored the nearby public health facilities and others preferred private facilities due to convenience, a shorter waiting time, a broader range of services, and better quality and reliability. Some also expressed distrust against free services, expressing concerns about potential low quality [16]. The typology of different types of provisions of non-government services is outlined in Table 6.

#### 3.2.2. Identified Solutions to Improve Service Availability

The aims of solutions to improve health planning in urban areas included the improved collection and use of data, improved vaccine forecasting, enhanced political commitment and financing for human resources and service delivery. The strategies identified the need to engage private healthcare providers, as there are more private providers in urban areas. This included a common recommendation to identify a model for immunization delivery between the government and private providers. Proposed models include contracted service delivery, referral from private to public services to immunization, and the need to include healthcare workers of private health facilities in immunization trainings. The recommendations and proposed interventions for country strategies are available in Table 7.

### 3.3. Quality of Service

#### 3.3.1. Identified Challenges to Service Quality

The main demand-side barriers in urban areas ranged from lack of information—poor communication on the importance of vaccination, location, and timing of sessions—to stronger deterrents, including misinformation and poor treatment by health workers. Further, the lack of information on the demographics of beneficiaries for tailored communications, attention to child-friendly services, and models of community mobilization were largely absent from program plans and diagnostics.

Costs related to seeking immunization services in urban areas included charges for services, commodities (cards, vaccines, diapers, paracetamol), transport, and lost wages. Despite existing policies protecting caregivers from costs for a free, public service, illegal charges were reported in numerous diagnostic studies. In Angola, a supervisor noted the constraint for “the population are the illegal charges, both for the vaccination cards and the vaccines” [17]. Similarly, in Bangladesh, where the government mandates that EPI vaccines be provided free of charge, one third of interviewed mothers reported the payment of a services charge, including at NGO-operated facilities [12]. In addition to paying for services, paying for cards and replacement cards was found across countries and regions including in DRC, Angola, Ghana, Haiti, and Uganda [10,13,17].

Long wait times for immunization sessions were cited across cities, ranging from only 30 min (Kenya) to 5 h (Haiti) [10,14]. Symptomatic of planning and financing issues, long waiting time was reported to impact care-seeking behavior and health worker interaction, while being also a deterrent for working parents, increasing opportunity costs for parents and the utilization of more efficient and privately operated clinics. In Ghana, wait times were cited by 24% as reasons for non-vaccination [13].

Respondents in Ghana cited being discriminated against for not having nice clothes to wear to the facility and 44% of respondents in the study noted the negative attitude of the provider as a discouraging factor [13]. In Sierra Leone, mothers and caregivers reported being hesitant to return to health facilities for fear of being “chastised, rebuked or insulted” if they brought the children late back to vaccination [18]. 

Working parents, particularly those employed in the informal sector, faced a choice to vaccinate or lose income required for daily subsistence. In Bangladesh, 17% of caregiver respondents reported being “too busy” to complete the full vaccination schedule [12]. In Somalia, it was reported that “low-income mothers were often too busy searching for food and other forms of income to prioritize health seeking services for their children” [8].

#### 3.3.2. Identified Solutions to Improve the Quality of Service

Recommendations and proposed strategies ranged from healthcare worker interpersonal training to the hiring of outreach workers from marginalized populations to improve engagement. Efforts to tailor the service-delivery model to working and busy caregivers included interventions such as a dedicated vaccination line and after-hours vaccination. The use of mobile phone networks to share information and vaccination reminders was proposed in more than seven countries. Child-friendly improvements to clinics was a common intervention to improve the quality of care and immunization experience. The recommendations and proposed interventions for country strategies are available in Table 8.

## 4. Discussion

The strengths of this study draw from the number of documents reviewed across a broad range of countries in different regions, encompassing more than 70 urban centers. Despite the variance in socio-economic realities between the settings, common challenges and solutions emerged which may be the starting point and reference for the design of effective urban immunization programs. Although several of the barriers and solutions identified could be valid across rural and urban populations, the findings were in line with the Gavi Urban Immunization Programming Guidance and the Equity Reference Group discussion paper on tackling immunization inequities in urban contexts, which outlines the following issues as unique to urban areas or exacerbated due to the urban context. In summary, these include the insufficient availability of public facilities, high population density and insufficient disaggregated data, seeking of healthcare across public and private facilities, a lack of formal addresses, insecurity, highly mobile populations, caregivers’ demands on time due to employment, complex community structures and a diverse population, requiring a tailoring of activities [19,20]. This analysis confirms these themes as critical barriers in achieving urban immunization equity, while highlighting issue for specific populations.

The findings for identified challenges due to social and economic determinants of health, including residents of informal settlements and migrants, are consistent with the literature, as documented in the systematic review by Crocker-Buque et al. [21] However, this review expanded potential populations to include homeless populations and internally displaced persons (IDPs) for consideration and prioritization in urban strategies. IDPs may have insecure residence, not know the location of key services, or have competing priorities, which may require a tailored approach. 

Results showed challenges related to the growth of private sector services and costs to caregivers, including the loss of income [22]. While these challenges were present in the majority of the cities covered by this research, there is little documented evidence on this issue in the literature, despite costs for private service and transport and loss of income, representing potentially significant barriers for poor urban populations [22]. The evidence shows caregivers may face loss of income or costs to attend an immunization session while the same caregivers receive little social mobilization and information on the benefits of the service to motivate attendance.

Challenges to immunization equity in the urban context largely link to appropriate health planning and the design of inclusive and accessible health services for populations of concern and working caregivers [23,24,25]. The number, location, and accessibility of immunization sites was inadequate across many studies and poor microplanning for outreach exacerbated issues of health access while affecting quality of care due to long wait times, overworked healthworkers, negative attitude of service providers, and poor vaccine stock availability. This was consistent with the existing literature [26,27]. Leveraging multisectoral approaches in urban contexts could help improve overall service delivery and quality [28]. 

The poor quality of services, rooted in the planning, financing, and execution of immunization sessions, in areas of rapid urban growth likely results in poor demand for immunization services in many urban poor areas. The specific causes of reduced demand linked to service delivery in urban poor areas are related to quality of care and include stock-outs, poor interaction with health workers, wait time, constrained hours, and cost. The recommendations for improvements in these areas include investment and prioritization, bringing children to already existing services in under-resourced areas, improving healthworker interaction and rebuilding the reputation of the immunization programs. These are consistent with the review by Oyo and Nelson et al. [29,30].

In some settings, there was a refusal of care to populations of concern such as migrants. The review showed health workers were not well represented in the studies and further evidence is needed to understand their work environment and challenges in providing care in urban areas and biases in serving populations of concern. The issues with quality of health worker interaction were consistent across the majority of studies and recommendations. However, as noted by Suphanchaimat, there are also constraints in resources available to health workers and incongruence of legislation and professional norms and ethics in provision of service to migrants [31].

Although the review presents new evidence to contribute to the evidence of urban immunization equity, the study is limited due to non-standardization of the methods in the documents included in the review. There was no uniform method to the 30 studies reviewing barriers and challenges to immunization equity which limited the analysis to a documentary review. Further, the availability of data from key areas such as gender was limited as the diagnostic tools used for most of the urban immunization studies sourced for this review did not include a robust methodology for gender analysis. As the documents included in the analysis were developed at the planning and strategy-development phase, the results only show proposed solutions and interventions, without data on which interventions may be effective. Finally, the analysis does not compare the urban and rural experience due to approach and similar available documentation specific to rural environments. 

The research identified areas for further inquiry. Additional gender research is needed to include complex issues such as working parents, the link between health services and daycare, building on recent analysis in this space [32,33]. The interventions mapped in the analysis are only proposed interventions, and the efficacy and impact of the interventions remains a key research gap [34]. The issue of cost barriers was present across contexts and represents a significant future area of research. In addition to service costs, the loss of income (opportunity cost), cost for commodities such as immunization cards or diapers and transport requires further study. Finally, the capacity and service quality of the private sector health service providers have not been extensively documented and evaluated as part of the diagnostics and strategies reviewed in this analysis. The development of models for the engagement of the private sector would benefit from a mapping and further analysis of existing contractual and operating arrangements, across countries. 

## 5. Conclusions and Recommendations

The combination of insufficient service delivery for immunization and poor quality of care, particularly for populations of concern shows the importance of tailored health investments and strategies in cities and provides evidence to explain the large number of zero-dose and under immunized children residing in urban contexts. The recommendations compiled through the review and developed uniquely by each country in their strategy development represent an important starting point for urban immunization programming to overcome barriers commonly identified in the urban context. Table 7 represents innovative strategies to improve urban immunization equity, proposed by countries. Evaluating the effectiveness of the interventions is an important next step to build the body of evidence for effective strategies. 

This research can help inform and improve guidance in the development of urban immunization strategies and presents additional research priorities needed to evaluate suggested interventions in addressing urban equity. A research agenda based on the gaps identified in the review and input from the urban immunization working group is presented in Table 9 and recommended by the authors for future studies. 

## Figures and Tables

**Table 1:** Research questions for urban immunization analysis.

Category	Summary	Research Questions
Urban environment	Overview of the context in urban poor settlements, and key data	What are the common and unique demographic features (high mobility, overpopulation, poverty, and socioeconomic conditions) associated with inequitable access to immunization in urban settlements?
Barriers to immunization: service delivery	Assessment of the health service facilities and the bottlenecks	What are the common and unique limitations of urban immunization service points impacting vaccine delivery?
Barriers to immunization: demand	Experience of caregivers and health service providers	What are the common and unique socioeconomic, behavioral, and practice-related factors affecting equitable access to immunization in poor urban settlements?
Recommendations and strategies for improvement	Identifying common and unique recommendations with the potential to ensure equitable immunization access in urban settlements	(1) What are the proposed interventions to improve service delivery and demand-side barriers to equitable immunization coverage in urban areas? (2) Which interventions have shown improved coverage in urban poor areas?
Areas of further study	Identifying the expected outcomes of the research and scopes for further implementation-related research agendas	What are the key knowledge gaps in (1) the study of barriers to improving equity in urban areas and (2) understanding the impact of interventions which require further study?

**Table 2:** Eligibility criteria for document review.

Category	Eligible	Ineligible
Time	2016–2020	Pre-2016, post-2020
Language	All languages	None
Types of document	Health system-strengthening applications, diagnostic reports, coverage and equity assessments, technical assistance plans, urban immunization strategies	Budget documents including application budget documents and costed components of the strategies
Geographic area	Gavi-eligible countries	Non-Gavi eligible countries

**Table 3:** Populations of concerns and associated barriers.

Populations of Concern	Documented Barriers
Migrants	Frequent address change, low standard of living
Refugees	Lack of legal recognition, limited access to public services, discrimination
Homeless populations	No permanent residence, limited income sources, illegal status in the city
Internally Displaced Persons (IDPs)	Lack of recognition, discrimination
Residents of informal settlements	Lack of legal recognition, limited access to public services, low standard of living

**Table 4:** Strategies identified to overcome social and economic determinants of health for populations of concern.

Tailored Interventions (Solutions)	Countries with Recommendations or Strategies
Mobile and outreach strategies (market, street, places of worship)	Nepal, Pakistan, Sierra Leone, DRC, Kenya
Improved training of health workers on interaction and information (AEFI), etc.	Afghanistan, CAR, Djibouti, Ghana, Nepal, Haiti, Indonesia, Kyrgyzstan, Pakistan, Senegal, Uganda, Kenya, Somalia, Bangladesh
Tracking of migrant families	Kyrgyzstan, Somalia
Recruitment of more female health workers	Afghanistan
Addition of community health workers or incentives, hiring of health volunteers from marginalized communities	Haiti, Sierra Leone, Afghanistan, Ghana, Myanmar
Use of data reviews for underserved areas	DRC, Ghana, Haiti
Engage religious leaders	Afghanistan, Somalia, Myanmar
Gender research on barriers	Afghanistan, Myanmar, Bangladesh
Monitor and remediate illegal payments	Angola, Bangladesh
Community engagement in planning, participating in, overseeing, and/or mobilizing services	Ghana, Kyrgyzstan, DRC, Kenya, Pakistan, Senegal, CAR, Haiti, Indonesia, Myanmar, Kenya

**Table 5:** Service-delivery challenges and associated barriers for caregivers.

Immunization Session Component	Service-Delivery Barriers	Challenge for Caregivers
Health worker	− Insufficient data on population − Insufficient supply − Poorly trained − Overworked − Poor monitoring and supervision − High turnover	− Poor interaction and discrimination by health worker − Long wait times − Adverse events (abscess, bleeding) − Refusal of vaccination due to child illness
Location of immunization site and availability hours	− Long distance to site − Replacement of public service by private and non-government providers − Security concerns for health workers	− Cost of travel, long travel time − Cost of cards or services − Loss of income for attendance of sessions during work hours − Security concerns for caregivers
Cold chain	− Non-availability of quality cold chain	− Lack of vaccines and quality, potent vaccines
Vaccines	− Poor forecasting due to insufficient information or allocation policy − Poor distribution of vaccines − Poor application of open-vial policy	− Frequent stock-outs reduce willingness of caregiver to return for immunization sessions, particularly when facing a combination of the above barriers

**Table 6:** Types of Service Provision in Urban Areas.

Type of Provider	Characteristics
Public	Services provided by the government
Private	Services provided by for-profit providers. These tend to fall into two categories: (1) private facilities for the highest wealth quintiles with a focus on quality and (2) private facilities which fill a gap in government services and tend to be less regulated.
Faith-based and not-for-profit private	Services provided by non-profit entities, usually subsidized by faith-based or non-governmental sources.

**Table 7:** Strategies identified to improve service-delivery availability and health planning in urban areas.

Tailored Intervention	Country with Recommendation or Strategy, Included
Electronic Immunization Registry	Bangladesh, Kenya, Indonesia, Uganda
GIS Mapping (and digital microplans)	DRC, Myanmar, Pakistan, Haiti, Uganda
Defaulter tracking	Uganda, Indonesia, Sierra Leone
Digital Temperature Monitoring	Somalia, Kenya
Establish new vaccine centers	Afghanistan, Bangladesh, Ghana, Kenya
Increase human resources	Afghanistan, Bangladesh, Afghanistan
Schedule appointments	Kenya, Indonesia, Ghana,
Ensure immunization offered, daily through the use of supervision and financing	Haiti, DRC, Pakistan, Indonesia, Kenya
Add vaccine stores and CCE availability	Nepal, Afghanistan, Bangladesh, CAR, Ghana, Indonesia, Sierra Leone, Somalia
Develop private sector strategy and/or engagement with FBO and CSOs	Pakistan, Afghanistan, Angola, Bangladesh, DRC, Ghana, Kyrgyzstan, Nepal, Uganda, Senegal
Strengthen vaccine management, reduce stock outs	Bangladesh, DRC, Haiti, Ghana, Sierra Leone, Somalia, Kenya
SMS and Whatsapp groups of healthcare providers for support and training	Haiti
E-training	Indonesia
Reinforce appropriate use of open-vial policy	Kyrgyzstan, Ghana
Engaging private sector HWs in EPI training	DRC, Indonesia, Nepal, Senegal
Household census, improved denominator	Afghanistan, DRC, Kyrgyzstan, Uganda, Angola, Sierra Leone
Strong link with antenatal care	Bangladesh
Hire or redistribute additional vaccination staff	Kenya, Senegal, Uganda
Establishment of unit dedicated to urban public health	Bangladesh
Service integration	Indonesia, Myanmar, Kenya
Improve financing for Immunization and PHC	Pakistan, Bangladesh, Haiti, Kenya,
Advocacy of leadership	Pakistan, Uganda, Haiti, Senegal, DRC
Development of actionable plans	Bangladesh, Uganda
Microplanning	DRC, Ghana, Afghanistan, Bangladesh, Indonesia, Somalia
Improve monitoring and supervision	Ghana, Haiti, Senegal, Kenya, Afghanistan, Angola, CAR, Indonesia, Pakistan

**Table 8:** Strategies identified to improve quality of service and demand for immunization.

Tailored Interventions (Solutions)	Country with Recommendations or Strategies
Rapid/dedicated vaccination line	DRC, Haiti
Expand hours (evening, weekend)	Ghana, Haiti, Somalia, Kenya, Uganda
Improved training of health workers on interaction and information (AEFI), etc.	Afghanistan, CAR, Djibouti, Ghana, Nepal, Haiti, Indonesia, Kyrgyzstan, Pakistan, Senegal, Uganda, Kenya, Somalia, Bangladesh
Use of mobile network infrastructure to promote RI communications (SMS, reminders, Whatsapp)	Bangladesh, Senegal, Angola, Afghanistan Ghana, Indonesia, Somalia
Improve communication on availability and location of services	Somalia, Myanmar
Develop child-friendly clinic, environment, including offering food at clinic	Kenya, Ghana
Awards for caregivers/child	Ghana, Kenya
School health curriculum or link	Afghanistan, Kenya, Bangladesh

**Table 9:** Recommendations for further research.

Recommendations for Further Research
The following research items were identified as part of the review; How private sector providers and civil society can be engaged and incentivized to deliver accessible and high quality immunization services, in a sustainable manner, including to marginalized populations? What are the estimated costs to caregivers for immunizing their child, the impact on uptake, and possible solutions? How can existing rural community health volunteer and social mobilization models be adapted successfully to the urban health space? What does it cost to deliver quality services and outreach to zero-dose and under-immunized urban communities? How can the governance structure in a city be engaged in the urban health and development space to ensure sustainable and city-owned immunization solutions? What are the gender barriers facing urban caregivers, children, and health workers? What are the challenges experienced by health workers in the public and private sectors in delivering immunization in an urban setting? What is the availability and type of cold chain most appropriate for urban contexts? How can social protection programs be leveraged and work with health programs to identify and reach marginalized groups?

## Data Availability

The country strategies and diagnostics are owned by Ministries of Health. No permission was sought to share this information more widely.

## Supplementary Materials

The following supporting information can be downloaded at: https://www.mdpi.com/article/10.3390/vaccines11071200/s1, Table S1: Coding Framework and Table S2: List of documents in scope of qualitative analysis of urban immunization studies.
