# Supplementary material for: Improving Equity in Urban Immunization in Low- and Middle-Income Countries: A Qualitative Document Review
Source: Vaccines (Basel). 2023 Jul 4;11(7):1200. doi: 10.3390/vaccines11071200 (PMC10386579; doi:10.3390/vaccines11071200)
Supplement: Supplementary file 1 [file vaccines-11-01200-s001.zip › vaccines-2418150-supplementary.pdf]

Table S1: Coding Framework

Themes	Sub-themes	Codes
1. **Urban Environment**	1. Demography	1. Population Density
		2. Living in informal settlements
		3. Number of care facilities
		4. Residency status
		5. Refugees
		6. Homeless Families
		7. IDPs
		8. Migrants & Migration
	2. Reason to visit healthcare points	1. Immunization
		2. Routine Checkup
	3. Type of service centers	1. Private (NGO and For-profit)
		2. Public
	4. Characteristics of health workers	1. Age
		2. Gender
		3. Experience
		4. Responsibility type
		5. Training
	5. Characteristics of the Caregiver	1. Age
		2. Gender
		3. Education
		4. Profession
		5. Socioeconomic Status
2. **Supply-side Barrier**	1. HW's knowledge on aspects of Immunization	1. Knowledge of vaccination
		2. Availability of guidelines
		3. Contraindications
		4. Lack of training for health workers
		5. Schedule
	2. Insufficient health planning	1. Lack of health workers
		2. Distance between service points
		3. Ill distribution of care facilities
		4. Lack of micro planning
		5. Lack of Immunization card
		6. Lack of EPI Management
		7. No/Poor Follow-up
		8. Lack of outreach activities
	3. Quality of Service delivery	1. Opening time
		2. Regularity
		3. Defaulter Tracing
		4. Waiting time
		5. Weekend facilities
		6. Inequality in services delivery
		7. Lack of waste disposal
	4. Vaccine availability	1. Vaccine Stockout
		2. Forecasting issues
		3. Vaccine Sources/Distribution
	5. Insecurity	1. Healthcare worker's insecurity

	6. Marginality	1. Caregivers and children with disability
		2. Working parents
	7. Out Pocket Cost	1. Other costs
		2. Vaccination cards cost
	8. Health records and data	1. Data Utilization
		2. Lack of disaggregated data
		3. Record books and missing records
	9. Cold Chain	1. Cold Chain Training
		2. Lack of Equipment
		3. Lack of functionality
		4. Protocols
		5. Lack of HR Plan
**3. Demand-side barriers**	1. Immunization Status and adherence to schedule	1. Imeliness of Immunization
		2. Immunization Status
		3. Zero Dose Children
	2. Information related Barriers	1. Not knowing Immunization schedule
		2. Fear of side effects
		3. Source of information
		4. Sick Children
		5. Underaged
		6. Language Barrier
	3. Interaction with health workers	1. Rude behavior
		2. Limited engagement with CBOs
	4. Maternal health related issue	1. Lack of antenatal care
		2. Reproductive responsibility
	5. Socioeconomic condition as a social barrier	1. Care-seeking behavior
		2. Childcare for other children
		3. Cost of travel for Immunization
		4. Poverty
		5. Religion/Belief Perception
		6. Caregivers without IDs
	6. Service quality	1. Lack of Bathroom availability
		2. Diapers as requirement for vaccination
		3. Exit survey
**4. Gender Barrier**	1. Female Healthcare worker	1. Facing discrimination
	2. Immunization responsibility	1. Household responsibility
		2. Ignorance accusation
		3. Socially constructed gender role imposing Immunization responsibility over women
	3. Physical and time barrier	1. Lack of mobility
		2. Lack of privacy for female caregivers at the service point
		3. Working mother time barrier use of alternative childcare
	4. Security	1. Female caregivers
		2. Female health worker
	5. Socioeconomic barrier	1. Economic vulnerability of female caregiver leading dropout
		2. Lack of decision making ability
		3. Limited/Lack of access to resources
		4. Low socioeconomic status

**5. Solutions**	1. Expansion of service availability	1. Extended hours
		2. Lack of Weekend facility
		3. Infrastructure
	2. Improve health planning	1. Balanced ratio between health workers and children
		2. Equitable distribution of service points
		3. Expand/improve cold chain availability & management
		4. Improve Funding
		5. Microplanning
		6. School-based approach
		7. Strategic Immunization day selection
		8. Strategic outreach points
		9. Improving EPI Monitoring
		10. Improving Data Management
		11. No stockout plan
	3. Improving caregiver and HW interaction	1. Creating service environment
		2. Stop being rude
	4. Multisectoral Collaboration	1. Stronger engagements of local CBOs
	5. Ongoing Interventions	1. Appointment Distribution
		2. Community Immunization Champion
		3. Community Sensitization
		4. Hiring Local HWs
		5. Immunization Buddy
		6. Monthly Stipend
		7. Refreshments (food)
		8. Waste Disposal and Infection Prevention
	6. Targeted demand generation	1. Community follow up
		2. Outreaches (Market, Street, Places of Worship)
		3. Politicians
		4. Printed Campaign (bus/billboard etc.)
		5. Radio communication
		6. Religious leaders
		7. TV
	7. Technological innovation	1. Digital Birth Registration
		2. Digital contact tracing for missed children
		3. Digital Follow up (SMS, WhatsApp, IVD, EIR)
		4. Digital Temperature Control
		5. Electronic Immunization Registry
	8. Trainings & Knowledge	1. Gender sensitive training for healthcare workers
		2. Social interaction training for HWs
		3. Technical knowledge training for HWs
	9. Gavi HSS	
6. Agenda for further research	1. Future scopes intervention research	
	2. Gaps in diagnostic methodology	

Table S2: Documents in scope of qualitative analysis of urban immunization studies

Country	Document	Type of Document	Date	Language
**Aghanistan**	Urban Immunization Strategy, Afghanistan	Strategy	2020	English
	Urban Immunization Strategy Presentation Slides, Afghanistan	Strategy - Presentation	2020	English
	Additional Health Systems Strengthening (HSS) Financing Proposal for Gavi	Investment Application	2019	English
	Afghanistan Additional HSS Funding- Independent Review Committee (IRC) Country Report	Application Review document	2019	English
**Angola**	Urban Immunization Situation Analysis	Study	2019	Portuguese
	Consultant Report of the urban immunization analysis	Study	2019	Portuguese
	Descrição Narrativa do plano de Acção de Luanda para a Estratégia de Imunização Urbana Plus (EIU+) 2020-25	Strategy	2015	Portuguese
**Bangladesh**	Child Immunization in Selected Urban Slums of Dhaka Bangladesh: Coverage and Associated Factors	Strategy	2019	English
	Additional Health Systems Strengthening (HSS3) Financing Proposal for Gavi	Investment Application	2019	English
**Central African Republic**	Projet de Stratégie urbaine de vaccination pour la ville de Bangui en 2018	Strategy	2019	French
	Projet de Stratégie urbaine de vaccination pour la ville de Bangui en 2018 - Presentation Slides	Strategy - Presentation	2018	French
	Health Systems Strengthening (HSS3) Financing Proposal for Gavi	Investment Application	2018	English
**Chad**	Equity Analysis	Study	2017	English
	Additional Health Systems Strengthening (HSS3) Financing Proposal for Gavi	Investment Application	2018	English
**Djibouti**	Urban Immunization Diagnostic Report and Costed Report	Study	2019	French
	Urban Immunization Strategy and Budget	Strategy	2019	French
**Democratic Republic of Congo**	Reaching Unvaccinated Children: Lessons Learned from JSI's Technical Assistance to the Government of the DRC for Improving Access and Ulitization of Routine Immunization Services	Study	2019	English
	Improving RI Service Delivery to Urban Poor in Kinshasa, Democratic Republic of Congo: Results ofSituational Analysis from JSI	Study	2018	English
	Rapport diagnostique : Analysis Situationnelle des Zones Urbaines Defavorisees de Limete et Kimbanseke a Kinshasa	Study	2018	French

	Stratégie et Activités Pour L'amélioration de L'Immunization en Milieu Urbain Défavorisé à Kinshasa, RDC	Study	2018	French
**Ethiopia**	Strengthening Ethiopia's Urban Program (SEUHP) From JSI	Study	2019	English
	Analysis of the Core Functions, Issues, and Challenges of Human Resource Management for Urban Health Extension Professionals	Study	2015	English
	Situational Analysis of Urban Sanitation and Waste Management	Study	2015	English
	Strengthening Ethiopia's Urban Health Program	Study	2015	English
**Ghana**	TA for Improving Immunization Service Delivery for Urban Poor in Ghana: Report of Situational and Barriers Analysis in Seven Urban Centers	Study	2018	English
	Improving Routine Immunization Service Delivery to Urban Poor in Ghana: Results of Situational Analysis	Study	2018	English
	12-Month Technical Assistance Workplan for Reaching the Urban Poor in Ghana	Strategy	2018	English
	JSI Technical Assistance to Ghana for Improving Immunization Service Delivery to the Urban Poor (Validation workshop ppt)	Strategy - Presentation	Unknown	English
	JSI Technical Assistance to Ghana for Improving Access to Immunization for urban poor (Inception meeting PPT)	Strategy - Presentation	Unknown	English
**Haiti**	Strengthening Vaccination Programs in Haiti's Urban Communities to End Vaccine Preventable Deaths- Presentation Slides from JSI	Strategy - Presentation	Unknown	French
	Lessons Learned- Strengthening Vaccination Programs in Haiti's Urban Communities to End Vaccine Preventable Deaths	Study	Unknown	English
	Assistance Technique pour Amélioration de Prestation de Services de Vaccination dans le Contexte de l'Aire Métropolitaine, Urbain Pauvre : Cité Soleil, Haïti	Strategy	2018	French
	Strategies for Improving Vaccine Coverage in Urban Poor Setting	Strategy	Unknown	English
**Indonesia**	Diagnostic Assessment on Immunization Service Delivery	Study	2019	English
	What Makes Vulnerbale Urban Populations Hard to Reach in Indonesian Cities?- From Universitas Indonesia and JSI	Study	2019	English
	Indonesia: Review of Policy Documents for Urbanization	Study	2019	English
	Strategies for Strengthening Immunization in the Urban Poor in Indonesia:	Strategy	2019	English

	Lessons Learned and Recommendations from South Jakarta, Central Jakarta and South Tangerang			
**Kenya**	Diagnostic Methodology Report: Report on Immunization Diagnostic Methodology for Nairobi and Kisumu Counties	Study	2019	English
	Additional Health Systems Strengthening (HSS2) Financing Proposal for Gavi	Investment Application	2019	English
	Developing Urban Immunization Strategy for Nairobi and Kisumu Counties - Documents for Best Practices and Lessons Learned	Strategy	2019	English
	Development of an Urban Immunization Strategy from PATH	Strategy	2019	English
**Kyrgyzstan**	Technical Assistance to the Government of Kyrgyzstan to Improve Immunization Service Delivery for the Urban Poor: Results of Situational Analysis and Recommended Next Steps	Strategy	2018	English
	Improving Routine Immunization Service Delivery to Urban Poor in Kyrgyzstan: Results of Situational Analysis	Study	2018	English
	Additional Health Systems Strengthening (HSS) Financing Proposal for Gavi	Investment Application	2019	English
	Kyrgyzstan Additional HSS Funding- Independent Review Committee (IRC) Country Report	Application Review document	2019	English
**Latin America (General)**	Regional Meeting to Share Lessons Learned to Improve Immunization Rates in Urban and Peri-urban Populations	Meeting Report	2018	English
**Myanmar**	Additional Health Systems Strengthening (HSS) Financing Proposal for Gavi	Investment Application	2019	English
**Nepal**	Summary Report of Assessment of Private Sector Immunization Services	Study	Unknown	English
	Health Systems Strengthening Application	Investment Application	2018	English
**Pakistan**	Survey on Status of Immunization in Urban Slums of Rawalpindi, Lahore and Multan of Punjab Province of Pakistan	Study	2016	English
	Micro Census Report on Status and Barriers to Immunization in the Urban Slums of Sindh, Karachi & Hyderabad	Study	2017	English
	Summary: Roadmap for Achieving Universal Immunization Coverage in Karachi, Pakistan 2019-2022	Study	2018	English
	Urban Immunization Brown Bag: Pakistan Experience - Presentation Slides	Study - Presentation	2019	English
	Additional Health Systems Strengthening (HSS) Financing Proposal for Gavi	Investment Application	2019	English
	Roadmap for achieving universal immunization coverage in Karachi Pakistan 2019-2022	Strategy	2018	English

**Senegal**	Plan de relance de la vaccination dans la région de Dakar: leçons apprises et perspectives- Presentation Slides	Strategy - Presentation	2017	French
	Plan Immunization en Milieu Urbain Senegal	Strategy	2017	French
	Strategie Urbaine pour la Vaccination au Senegal	Strategy	Unknown	French
	Additional Health Systems Strengthening (HSS) Financing Proposal for Gavi	Investment Application	2019	French
**Siera Leone**	Immunization Coverage and Equity Assessment	Study	Unknown	English
	Additional Health Systems Strengthening (HSS) Financing Proposal for Gavi	Investment Application	Unknown	English
**Somalia**	Assessing Immunization Service and Delivery in Three Major Urban Somali Cities	Study	Unknown	English
	Additional Health Systems Strengthening (HSS) Financing Proposal for Gavi	Investment Application	2018	English
**South Sudan**	National Expanded Program on Immunization multi-Year Plan 2018-2022	Strategy	2018	English
	Additional Health Systems Strengthening (HSS) Financing Proposal for Gavi	Investment Application	2018	English
**Uganda**	Uganda Immunization Equity Assessment Report, September 2016; Communities and Districts Affected by Immunization Inequities	Study	2016	English
	Evaluation of the Drivers of Urban Immunization in Uganda: A Case Study of Kampala City May 2020	Study	2020	English
	Additional Health Systems Strengthening (HSS2) Financing Proposal for Gavi	Investment Application	2019	English
	From Response to Resilience: Working with Cities and City Plans to Address Urban Displacement (Lessons from Amman and Kampala) - the International Rescue Committee	Study	2018	English
	Welcoming Kampala: Achieving Refugee Integration & Building Urban Resilience - From the International Rescue Committee	Study	2017	English
